# Reproductive development of dairy heifers in an integrated livestock-forest system during the summer

**DOI:** 10.1590/1984-3143-AR2023-0100

**Published:** 2023-10-27

**Authors:** Hugo Rocha Sabença Dias, Agostinho Jorge dos Reis Camargo, Gabriela Ferreira Oliveira, Anderson Moreira Mourão, Naiara Zoccal Saraiva, Luiz Sérgio de Almeida Camargo, Marcelo Dias Müller, Carlos Eugênio Martins, Luiz Altamiro Garcia Nogueira, Felipe Zandonadi Brandão, Clara Slade Oliveira

**Affiliations:** 1 Faculdade de Veterinária, Universidade Federal Fluminense, Niterói, RJ, Brasil; 2 Embrapa Gado de Leite, Juiz de Fora, MG, Brasil; 3 Universidade Federal Rural do Rio de Janeiro, Seropédica, RJ, Brasil

**Keywords:** heat stress, reproductive development, integrated systems, bovine

## Abstract

This study aimed to assess the cortisol, body and reproductive development of prepubertal Holstein and Holstein-Gir ¾ heifers at 27 months of age maintained in an integrated livestock-forest (ILF) system for 60 summer days compared to the monoculture system in full sun (FS). The ILF system promoted changes (*P*=0.02) in the cortisol levels of Holstein-Gir ¾ heifers and did not affect weight gain in any of the breed groups studied. Animals in ILF system presented a lower (*P*=0.006) vulvar development for the rima height parameter and similar for the vulva width parameter. The ovarian follicular population of Holstein-Gir ¾ heifers in the ILF system was lower (*P*=0.004); however, for the Holstein heifers, no statistical difference was found, and numbers were higher (*P*=0.08) in the ILF system. None of the other ovarian parameters studied had any changes, and we also found important racial differences. Weight gain (*P*=0.003), vulvar development (*P*<0.001), and mean follicular size (*P*=0.008) were higher in the Holstein-Gir ¾ animals. Based on such results, the effect of the ILF system at 27 months of age on stress and reproductive parameters in the Holstein breed is considered positive, although negative effects have been detected on reproductive parameters in the Holstein-Gir ¾ breed.

## Introduction

The system of Livestock-forest integration (ILF) is a sustainable production model that brings together livestock and forest activities in the same area, in a rotating and consortium way to improve soil exploitation and reduce environmental impacts (Almeida et al., 2021; Landholm et al., 2019). Such a premise is based on the reduction of carbon dioxide and methane emissions, fixation of nutrients in the soil, rotation of areas, erosion mitigation, and pests reduction, thus presenting applicability to different types of environments (Peterson et al., 2020). Furthermore, it is aligned with the national extensive and semi-extensive rearing method, which seeks lower production costs linked to favorable conditions for the development of dairy heifers (Costa et al., 2013).

Integration systems can increase biomass production, allowing up to eight times more carbon storage than conventional forage monoculture systems (López-Santiago et al., 2019), which reduces the environmental damage caused by extensive and semi-extensive livestock systems due to fewer new areas open for exploration, in addition to mitigating enteric methane emissions by ruminants (Vermeulen et al., 2012).

In addition to the aformentioned economic and environmental benefits, integrated systems can improve the thermal comfort of animals exposed to high temperatures, especially for breeds that are less adapted to the tropical climate, such as the Holstein. There is clearly less rumination in animals allocated in a system without shading compared to the ILF system, thus indicating that the ILF systems have a greater potential for animal comfort (Giro et al., 2019). The tree component of the ILF system reduces the transmission of solar radiation in forages and animals (Oliveira et al., 2018) and its thermal load is 22% lower than in areas without shade, implying fewer hours of thermal stress during the day (Pezzopane et al., 2019). Animals move less in shaded systems since they feel no thermal discomfort (Améndola et al., 2019), which causes them to have a cohesive behavior and spend more time resting (Broom et al., 2013).

Heat stress is considered a limiting factor for the development of dairy heifers raised in a semi-extensive system (Sartori et al., 2002). Furthermore, it can also affect cortisol concentrations in dairy herds (Lyimo et al., 2000). Cortisol is associated with the inhibition of antibodies, the production of lymphocyte activating factors, and the compromising of the immune system (Munck et al., 1984).

Bovine females exposed to high temperatures spend more energy for homeothermy maintenance and their reproduction is damaged (Berman, 2011). Oocyte and embryo quality is affected when females are exposed to temperatures above 39.1 °C for a prolonged period, increasing oxidative stress (Hansen, 2019). According to Gwazdauskas et al. (1973), adding 0.5 ºC above the physiological temperature of the uterus may reduce fertility and cause hormonal changes. Stress also affects growth and delays the puberty onset (Cooke et al., 2019).

Currently, about 80% of the milk produced in Brazil derives from the Girolando breed (19), which comes from Gir and Holstein breeds. Holstein-Gir ¾ animals are adapted to tropical conditions and are more tolerant to high temperatures and ectoparasites (Ledic et al., 2002), a genetic background that can affect thermal stress, thus justifying the inclusion of crossbred animals in our study.

The study aimed to assess the influence of the ILF system on thermal stress and reproductive development of prepubertal dairy heifers.

## Methods

All procedures involving animals were approved by the Embrapa Dairy Cattle Ethics Committee in animal experimentation (CEUA EGL, 7374130921).

### Experimental area and animal management

We carried out the study at the Embrapa Dairy Cattle, in the Santa Monica Experimental Station – Valença (21°35”S, 43°51”W; 435 meters), Rio de Janeiro State, Brazil, of the Innovation Center in Agriculture Sustainable Intensification (NISA). The area encompasses four hectares on a smooth undulating terrain with an average slope of 20%. Two hectares were destined for the forestry and pastoral system of livestock-forest integration (ILF), and other two hectares for the single pasture system in full-sun (FS) monoculture. The experiment involved an average temperature of 23.5ºC, a maximum temperature of 35ºC, a minimum temperature of 14.9ºC, relative humidity of 96% (±13.81%), and precipitation of 54mm^3^ (±2.54mm^3^). The ILF system was implemented in November 2019 by introducing a clone of a hybrid of *Eucalyptus urophylla* S. T. Blake x *Eucalyptus grandis* W. Hill ex Maiden, (clone 1407). The trees were planted in contour lines and simple lines with a spacing of 25 meters. Within the lines, the trees were spaced at two meters, totaling a planting density of 200 trees per hectare and a basal area (1) of 1.33 m^2^ ha-1 at 27 months of age. Trees promoted shading throughout the system at some point in the day. *Brachiaria decumbens* corresponded to the grass used. The animals were allocated at the proportion of 1.8ha/animal, with *ad libitum* consumption. Each system was divided into three areas used for rotational management and determined by the grass height: 50 cm at the entrance of the animals and 20 cm at the exit. Mineral salt and 2kg portion per animal/day were offered.

### Experimental design

The study assessed the effects of the integrated forestry livestock (ILF) and full sun (FS) systems on cortisol levels and body and reproductive development Holstein and Holstein-Gir 3/4 heifers during the summer. For such a purpose, we used 32 heifers (8 per group) aged between 14 and 18 months divided into the following four experimental groups: Holstein-Gir ¾ in ILF system, Holstein-Gir ¾ in FS system, Holstein in ILF system, and Holstein in FS system. The animals were assessed every 14 days for serum cortisol levels and body weight, weekly for vulva length, and every ten days for ultrasound examination of the reproductive tract. Experimental procedures were carried out during the summer, between December and February, and only the cortisol analyses were performed between December and January. Genital evaluation was performed before the study to confirm the absence of corpus luteum formation.

### Cortisol dosage

Plasma was collected and stored at -20°C. Cortisol concentrations were determined by radioimmunoassay (Cortisol Coated Tube Kit Immuchem; MP Biomedicals, LCC Diagnostics Division, USA). The sensitivity factor and intra-assay coefficients were 0.17 ng/mL and 11%, respectively, as described by Lemes et al. (2021).

### Weight gain

The weighing was performed at an interval of fifteen days in four measurements in total. Mean weight gain was calculated based on the following formula: GMD= final weight – initial weight/ (weighing interval days).

### Vulva length

External genitalia was measured using a universal caliper (Digimess, São Paulo, Brazil), as follows: vulva width measured from the lateral edges from the rima midpoint at 90º (angle) and rima between the dorsal and ventral commissure, as described by Maculan et al. (2018).

### Ovarian follicle population

The reproductive development was assessed through ultrasound (Mindray DP2200®, 7.5MHz transrectal transducer) every twelve days, from December to February, with five assessments in total. The records included the number of follicles between 3 and 8 millimeters (mm), the number of follicles larger than 8 mm, the size of the largest follicle, and the presence of corpus luteum.

### Statistical analysis

Cortisol levels were distributed differently in the FS racial group and were analyzed separately for the Holstein-Gir ¾ and Holstein breeds, being converted into the Holstein-Gir ¾ group by Johnson. ANOVA and Tukey tests compared the means of the FS group. Both the day of data collection and the system used were considered factors.

Vulva width, rima height, and the number of follicles from 3 to 8 millimeters were transformed by Box-Cox. ANOVA and Tukey tests compared the weight, size of the largest follicle, transformed vulva width, rima height, and the number of follicles from 3 to 8 millimeters means of the FS groups. Both the breed and the system used were considered factors.

A binary logistic regression compared the incidence of follicles larger than 8 millimeters in the FS groups considering the breed, day of data collection, and system used as factors.

## Results

Each breed has a separate graphs to highlight the effects of the ILF system individually. The statistical analysis considered the factor of breed and the results are described in the text.

### Cortisol levels

**Figure 1:**
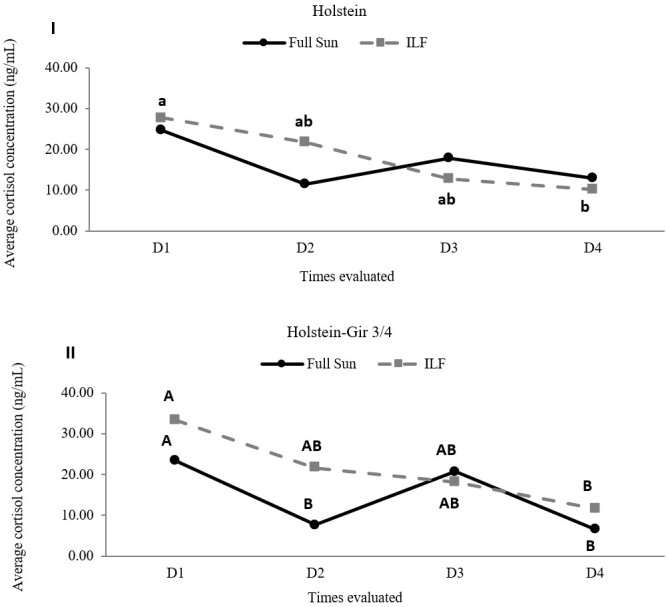
Evaluation of cortisol (ng/ml) levels in heifers maintained in the ILF and FS systems during the summer. Graph I shows cortisol levels in Holstein animals and graph II shows cortisol levels in Holstein-Gir 3/4 animals. Distinct lowercase letters indicate statistical difference between the collections for the ILF system and capital letters for the FS system. In graph II, there was no statistical difference in the Tukey test between the collections in the FS system. D1 = time zero; D2 = fourteen days; D3 = twenty-eight days; D4 = forty-two days; ILF = Livestock-forest Integration; FS = Full Sun.

Initially, we compared the cortisol levels in the animals of each breed, which showed no individual influence either for the Holstein (*P*=0.73) or the Holstein-Gir ¾ (*P*=0.27) breeds. The cortisol levels in the Holstein animals were similar (*P*=0.59) in both systems, ILF and FS (Figure 1.I). In this breed, lower (*P*=0.002) cortisol levels appeared throughout the experiment (data collection factor) with a statistical difference in the post-test (Tukey) only for the ILF system. There was no (*P*=0.17) interaction between the system factors and the day of data collection.

The Holstein-Gir 3/4 racial group showed higher (*P*=0.02) cortisol levels in the ILF system, and lower (*P*<0.001) cortisol levels throughout the experiment (day of data collection factor) in both systems (ILF and FS) (Figure 1.II). There was no (*P*=0.23) interaction between the system factors and collection date.

### Weight gain

**Figure 2:**
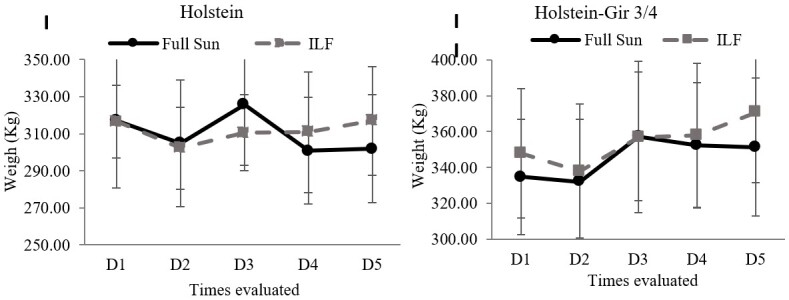
Live weight of heifers kept in the ILF and FS systems during the summer. The graph I shows the weight of the Holstein animals and graph II shows the Holstein-Gir 3/4 animals. D1 = time zero; D2 = fifteen days; D3 = thirty days; D4 = forty-five days; D5 = sixty days; ILF = Livestock-forest Integration; FS = Full Sun.

At the beginning of the experiment, the Holstein heifers had mean live weight of 316.50±19.57 (ILF) and 317.13±36.32 (FS), while the Holstein-Gir ¾ had 347.88±36.13 (ILF) and 334.75±32.25 (FS). Weight did not differ between the systems (Figure 2); however, different weight values (*P*<0.001) were found between the breeds throughout the experiment. There was no interaction between the system and breed factors (*P*=0.9).

**Figure 3:**
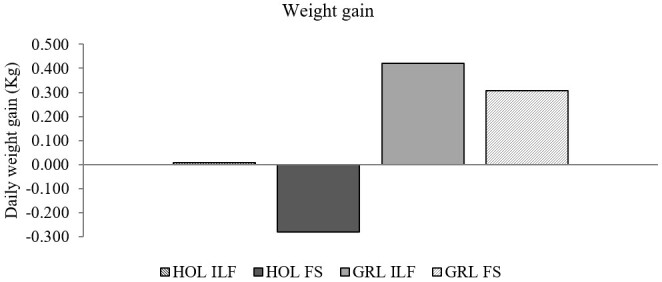
Average daily weight gain in heifers maintained in the ILF and FS systems during the summer. The graph shows weight gain in Holstein and Holstein-Gir ¾ animals. Data were analyzed by ANOVA considering two factors, race and system. There was no difference between the systems; the races presented different behaviors (p=0.003). There was no interaction between the factors (p=0.57). ILF = Livestock-forest Integration; FS = Full Sun.

As for weight gain, the system did not show to have any effect (*P*=0.20) on the breeds (Figure 3). The Holstein-Gir 3/4 animals showed higher weight gain (*P*=0.003) than the Holstein’s, which lost weight during the experiment. There was no interaction between the system and breed factors (*P*=0.57).

### Vulvar development

**Figure 4:**
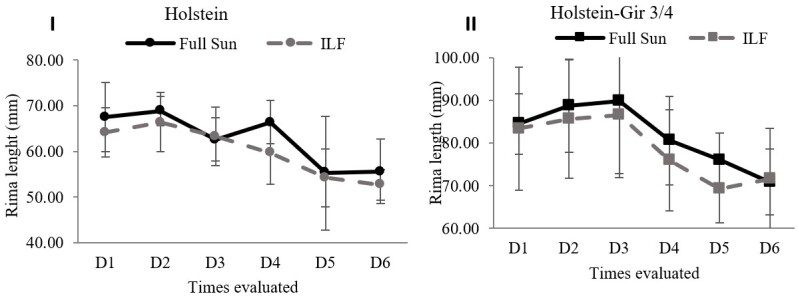
Rima length measurements in heifers maintained in the ILF and FS systems during the summer. Animals were kept in the systems for three months during the summer (DEC-FEB) and evaluated weekly. Graph I shows the rima length in Holstein animals and graph II of Holstein-Gir 3/4 animals. The graphs show unprocessed data. D1 = time zero; D2 = seven days; D3 = fourteen days; D4 = twenty-one days; D5 = twenty-eight days; D6 = thirty-five days; ILF = Livestock-forest Integration; FS = Full Sun.

The assessments were performed weekly (six days of collection). The rima height differed between the systems, with higher values (*P*=0.006) in the full-sun system (Figure 4). Differences were also identified between the breeds (*P*<0.001). There was no interaction between the system and race factors (*P*=0.65).

**Figure 5:**
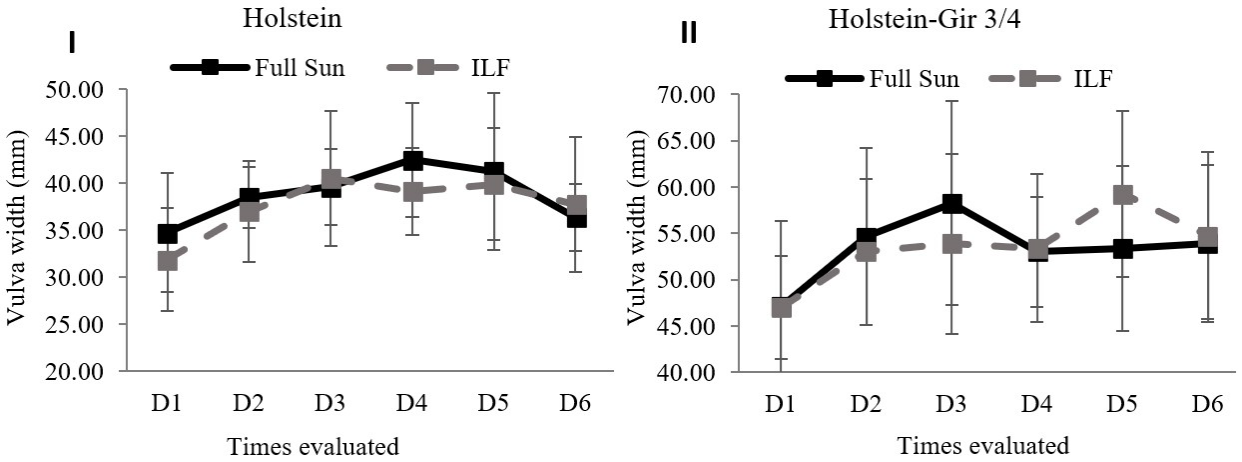
Development of vulva width in heifers maintained in ILF and FS systems during the summer. Graph I shows vulva width in Holstein animals and graph II in Holstein-Gir 3/4 animals. The graphs show unprocessed data. D1 = time zero; D2 = seven days; D3 = fourteen days; D4 = twenty-one days; D5 = twenty-eight days; D6 = thirty-five days; ILF = Livestock-forest Integration; FS = Full Sun.

The vulva width assessment found no difference (*P*=0.39) between the systems (Figure 5). The breeds presented different behaviors (*P*<0.001) and the averages were higher in the Holstein breed. There was no interaction between the system and race factors (*P*=0.32).

### Ovarian development

The animals started the prepubertal experiment (no corpora lutea was identified in either of the subsequent assessments with an interval of 15 days). At the end of the experiment, 43.75% of the Holstein-Gir ¾ heifers and only 31.25% of the Holstein heifers had reached puberty. The system had no effect on the distribution. Based on the small number of animals, this statistical analysis was not considered.

**Figure 6:**
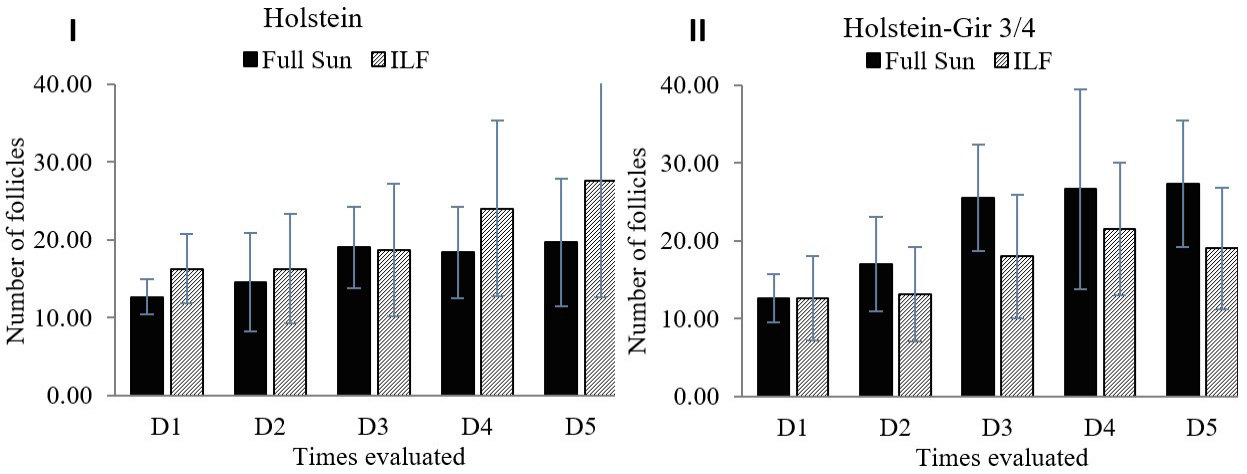
Evaluation of number of follicles in heifers maintained in the ILF and FS systems during the summer. The graph I shows the follicle count in Holstein animals and graph II in Holstein-Gir 3/4 animals. D1 = time zero; D2 = ten days; D3 = twenty days; D4 = thirty days; D5 = forty days; ILF = Livestock-forest Integration; FS = Full Sun.

The assessment of the number of follicles between 3 and 8 mm revealed an interaction (*P*=0.004) between the factors of system and race; therefore, the analyses were performed separately for each breed. The Holstein breed showed a numeric increase (*P*=0.08) to increase the number of follicles only in the ILF group (Figure 6.I). The Holstein-Gir ¾ showed a higher number (*P*=0.01) of follicles for the FS system (Figure 6.II).

**Figure 7:**
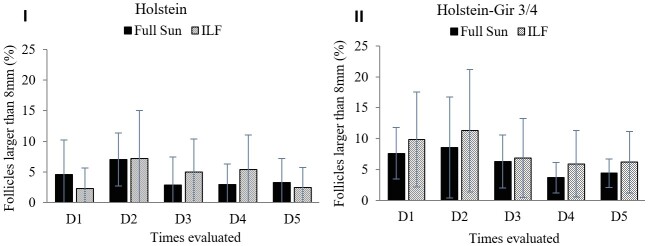
The graph shows mean percentage among animals of follicles larger than 8 mm in heifers maintained in the ILF and FS systems during the summer. The graph I shows the percentage of follicles larger than 8 millimeters in the Holstein breed and graph II in Holstein-Gir 3/4 animals. D1 = time zero; D2 = ten days; D3 = twenty days; D4 = thirty days; D5 = forty days; ILF = Livestock-forest Integration; FS = Full Sun.

The chance of occurring follicles larger than 8mm was similar (*P*=0.73) between the systems and the races (*P*=0.18) (Figure 7). The incidence corresponded to percentages of the ovarian follicular population of 0 and 25%, similarly to the Holstein and Holstein-Gir ¾ groups.

**Figure 8:**
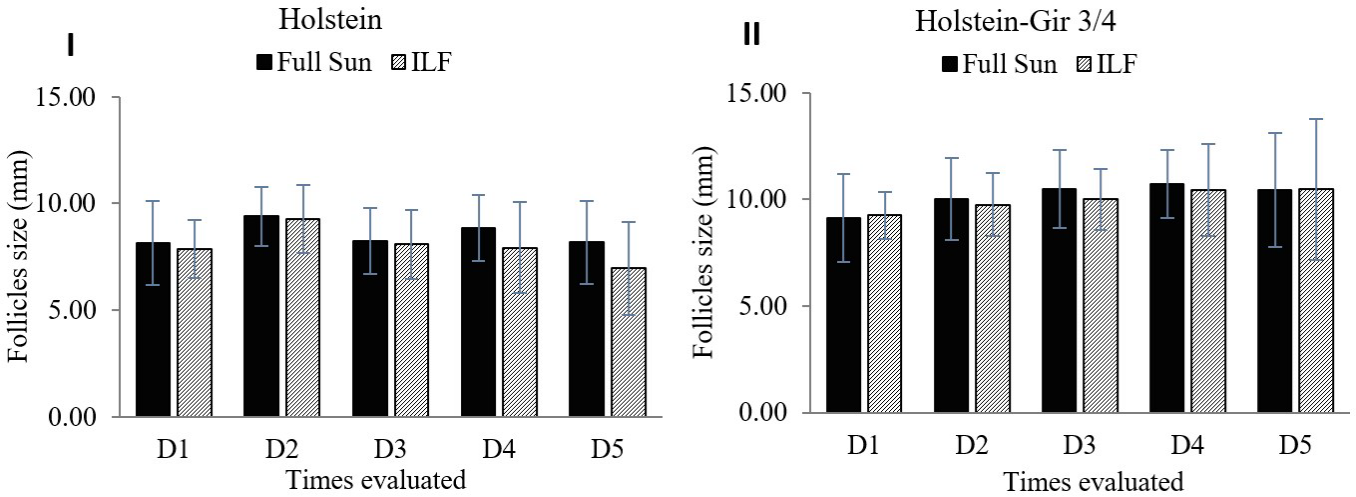
Size of the largest follicle in millimeters in heifers maintained in the ILF and FS systems during the summer. Graph I shows the averages of Holstein and the graph II shows Holstein-Gir 3/4 animals. D1 = time zero; D2 = ten days; D3 = twenty days; D4 = thirty days; D5 = forty days; ILF = Livestock-forest Integration; FS = Full Sun.

The analysis of the average size of the largest follicle indicated that the system exerted no effect (*P*=0.17) (Figure 8). The Holstein animals showed a lower (*P*=0.008) mean follicular size than the Holstein-Gir animals ¾. There was no interaction between the system and breed factors (*P*=0.13).

## Discussion

Seeking sustainable production models and animal comfort is a strong trend in livestock. Integrated livestock-forest systems represent important alternatives to offset methane emissions, reduce the carbon footprint, and mitigate heat stress in dairy cattle raised in tropical countries. Herein, we assessed the short-term development of dairy heifers in an integrated livestock-forest system during the summer, focusing on stress and reproductive development. In this context, we present the following main findings: i) the ILF system influenced the levels and pattern of cortisol in dairy heifers, ii) the ILF system did not interfere with the weight gain of dairy heifers, and iii) the ILF system exerted some effect on the reproductive development, positive in the Holstein females and negative in the Holstein-Gir ¾ females.

The ILF system did not influence the cortisol concentrations of the Holstein heifers. In turn, the cortisol levels slightly higher in the Holstein-Gir ¾ animals kept in the ILF system. The lower cortisol levels in cattle under heat stress has been associated (Christison and Johnson, 1972; Ronchi et al., 2001) with an adaptive mechanism resulting from the reduction of adrenocortical activity under conditions of heat stress. Therefore, our results suggest greater heat stress in the Holstein-Gir ¾ animals kept in the full-sun system. In turn, the similar cortisol levels between the systems in the Holstein breed may suggest that shading was not sufficient to attenuate heat stress in this breed. Both breeds had lower cortisol levels throughout the summer, as expected due to the adaptive mechanism discussed. However, in the Holstein breed, such a behavior was statistically significant only in the ILF group, suggesting that the system has a positive effect on the animals’ progressive adaptation to heat.

Neither live weight nor weight gain differed between the systems. As trees develop over the years, there is concern that the ILF system might interfere negatively with animal weight gain, due to the increase in shading promoted by the trees, consequently bringing a possible negative effect on grass quality (Paciullo et al., 2014). This study assessed a system of 27 months of age and a density of 200 trees ha-1, and chosen based on the positive influence on animal comfort parameters (Oliveira et al., 2018). Thus, the similarity between ILF and FS systems can be considered a positive finding. It is worth mentioning that the basal area of the system varies over time due to the size of the tree canopy and its growth, affecting environmental factors, microclimate, carbon sequestration, and light incidence (Jose, 2009), and possibly the animal performance in the system.

The Holstein animals ended up losing weight during the experiment, while Holstein-Gir 3/4 animals showed an increase in live weight. The genetic influence of *Bos indicus* might have affected weight gain since these animals gain more weight in tropical regions than the *Bos taurus* animals (Fontes et al., 2007). The Girolando breed is adapted to tropical climates (Souza et al., 2019) and other studies have demonstrated that their permanence in integrated systems resulted in satisfactory weight gain (Lemes et al., 2021; Paciullo et al., 2009).

There was a negative effect on the rima height of heifers allocated in the ILF system. Vulvar development reflects reproductive maturity in cattle since vulva length parameters are associated with follicle count, the hormonal incidence of estrogen, and proximity to puberty (Mesquita et al., 2016; Scheetz et al., 2012). Such an effect is possibly associated with the lower incidence of light in the ILF system, considering that luminosity positively interferes with reproductive parameters and puberty onset (Carvalheira et al., 2021; Hansen et al., 1983; Rius et al., 2005). Reinforcing this hypothesis, the Holstein-Gir ¾ showed a higher number of follicles (3 to 8mm) for the FS system.

However, even though the data presented suggest that the low ILF luminosity might have affected reproductive parameters, puberty occurred in a similar proportion in both systems. Thus, the shading system proved to be adequate to the way that favored the reduction of stress of these animals and energy reduction for thermal homeostasis.

Still, despite the negative influence of shading, the ILF system might benefit the number of competent follicles in the Holstein animals. This result suggests that for this breed, which is more sensitive to heat stress, the negative effect of the reduction in the light provided by the system did not overlap the positive effect of thermal comfort of shading, favoring the development of the reproductive system of animals kept in the ILF.

The Holstein animals (*Bos taurus*) are expected to enter their reproductive life earlier than animals with *Bos indicus* composition (Chelikani et al., 2003; Nogueira, 2004). However, the Holstein animals had a reduction in vulvar parameters due to the presence of smaller follicle. This difference concerning the Holstein-Gir 3/4 animals may reflect the lower weight of these animals. Loss of weight interferes with leptin concentration and has a negative effect on follicular maturation and development (Bruinjé et al., 2021). In addition, heat stress affects maturity, negatively influencing respiration rate and dry matter intake (Wang et al., 2020).

We must be aware of limitations in the present study that may have affected the results found, such as: short period of maintenance of the animals in the systems and period of life in which the animals were (peripubertal, in two distinct breeds). It is also important to emphasize that the microclimates in the different evaluated systems were not monitored, which did not allow to determine the effects of the systems on the average temperatures of the environment.

## Conclusion

We conclude that the studied integration-livestock-forestry system is suitable for the maintenance of dairy heifers. Its short-term effects during the summer provided cortisol modulation and breed-dependent effects on reproductive development, which do not seem to affect the puberty rate of the animals. The system represents an opportunity for greater economic use of land, provind the animals with greater comfort and potentially reducing the carbon footprint of dairy systems.

## Data Availability

The data sets generated during the current study are available from the corresponding author on reasonable request.
